# Authentication Based on Non-Interactive Zero-Knowledge Proofs for the Internet of Things

**DOI:** 10.3390/s16010075

**Published:** 2016-01-07

**Authors:** Francisco Martín-Fernández, Pino Caballero-Gil, Cándido Caballero-Gil

**Affiliations:** Departament of Computer Engineering, University of La Laguna, 38271 La Laguna, Tenerife, Spain; pcaballe@ull.edu.es (P.C.-G.); ccabgil@ull.edu.es (C.C.-G.)

**Keywords:** non-interactive zero-knowledge proof, authentication, mobile *ad hoc* network

## Abstract

This paper describes the design and analysis of a new scheme for the authenticated exchange of confidential information in insecure environments within the Internet of Things, which allows a receiver of a message to authenticate the sender and compute a secret key shared with it. The proposal is based on the concept of a non-interactive zero-knowledge proof, so that in a single communication, relevant data may be inferred to verify the legitimacy of the sender. Besides, the new scheme uses the idea under the Diffie–Hellman protocol for the establishment of a shared secret key. The proposal has been fully developed for platforms built on the Android Open Source Project, so it can be used in any device or sensor with this operating system. This work provides a performance study of the implementation and a comparison between its promising results and others obtained with similar schemes.

## 1. Introduction

The Internet of Things (IoT) is a trending concept that arises from the need to monitor and interconnect billions of information devices, typically equipped with sensors, actuators, microprocessors, communication interfaces and/or power sources. New challenges related to wireless security appear in IoT because the most common medium used for communication among hyper-connected devices is wireless. Thus, since most IoT objects have limited processing power, finite memory and low battery life, new lightweight cryptographic algorithms, in terms of processing and memory requirements, are necessary to obtain the efficiency of end-to-end communication and applicability to low-resource devices [1].

With the advent of increasingly powerful technology that is reduced in size and weight, the security level of the cryptographic schemes that are used in wireless communications has been increasing due to the rise of new algorithms based on harder mathematical problems, such as elliptic curves. In particular, the evolution of networks towards IoT has implied that this process is now accelerated.

Due to the emergence of this new paradigm of interconnected objects, where the physical dimension mimics the logical dimension, it is necessary to encode more than 100,000 million objects [2], because each person is surrounded by approximately 3000 objects. As previously mentioned, a key aspect to consider is the way of communication between these objects. Thus, the mobile nature and small size of many of these devices that form the IoT usually imply that such a communication is wireless and that the resulting network is established as a mobile *ad hoc* network (MANET), which is a network composed of mobile devices, wirelessly connected, and generally characterized by properties of auto-configuration.

Each device that is a member of a MANET can move freely, which involves that the link conditions between different devices can be continuously changing and requires that any node can act as a router for the communications of other nodes. Another important aspect of these networks is that, in general, they can operate independently or be connected to the Internet. When a member of the network is connected to the Internet, usually the network enables Internet access to the other devices that do not have a direct Internet connection.

Regarding security, in MANETs, many types of threats exist that can affect their use. Two of the most dangerous attacks are against the security of wireless communications through denial-of-service and man in the middle (MitM) attacks.

The proposal described in this work aims to be applied mainly to IoT devices used in mobile environments, such as vehicles, for example. In particular, this work proposes the design and implementation of a new lightweight cryptographic scheme to enable secure wireless communications in a MANET. It describes the design and analysis of a new scheme based on a non-interactive zero-knowledge proof for the authenticated exchange of confidential information in IoT, which allows a receiver node to authenticate the sender and to compute a shared secret key from the received message.

This paper is organized as follows. Section 2 introduces a few related works. Some necessary preliminaries are defined in Section 3. Then, a non-interactive zero-knowledge proof authentication scheme is proposed in Section 4. Several applications of the proposed scheme in sensors and IoT are briefly described in Section 5. Security aspects of the proposal are analyzed in Section 6. The performance evaluation is presented in Section 7, followed by a comparative analysis given in Section 8. Finally, Section 9 closes the paper with a few conclusions.

## 2. Related Works

Security in the Internet of Things is a primary factor. As a proof of that, the new operating system that Google has introduced for the Internet of Things, called Brillo, follows the “secure by default” paradigm. The work presented in [3] explains perfectly the major security threats to the Internet of Things, classified into three main categories:

- Related to the physical nature of smart objects: cloning of smart things by untrusted manufacturers, malicious substitution of smart things during installation, firmware replacement attack and extraction of security parameters since smart things may be physically unprotected.
- Related to intercommunication between smart objects: Eavesdropping attack if the communication channel is not adequately protected, man-in-the-middle attack during key exchange, routing attacks and denial-of-service attacks.
- Related to sensible data processed by the smart objects: Privacy threats.

If secure communication is established over insecure channels through symmetric cryptography, the secure pre-distribution of secret keys is one of the most essential tasks. This topic has attracted much attention among researchers [4,5] throughout the years. However, the best-known system for the secure distribution of secret keys via insecure channels continues being the Diffie–Hellman scheme [6], which allows two users to compute a shared secret key from two secret numbers and a public exchange of information, thanks to the discrete logarithm problem. Such an algorithm does not include user authentication, which leads to the possibility that am MitM attack can be launched. In order to avoid it, asymmetric cryptography and public key certification can be used. The present work faces the problem of the secure distribution of secret keys based on the idea under Diffie–Hellman scheme, but including authentication and using graph theory problems instead of the discrete logarithm problem.

In the case of MANETs, different proposals exist to protect communications, which are based either on secret-key cryptography or on public-key cryptography, like, for example, [7] and [8].

On the one hand, the security level of many symmetric schemes is high, but their major drawback is the difficulty of the pre-distribution of shared secret keys. In an environment like that of MANETs in the IoT [9], the assumption regarding the existence of a fully-secure channel to transmit the symmetric keys is very difficult to achieve. In addition, if the MANET is large and based only on symmetric cryptography, the number of secret keys that would be required is very high. Thus, as previously ]mentioned, to solve the problem of secure secret key distribution, asymmetric cryptography can be used.

On the other hand, public-key cryptography offers the capability of a digital signature scheme [10] if the sender decrypts with its private key and the receiver encrypts with the public key of the sender. Through a digital signature, authentication of both the sender and message, e.g., identification and integrity, can be guaranteed. Interestingly, the ability to use digital signatures that public-key cryptography provides is exactly what solves the main challenge it presents, which is the need to establish trust in public keys to prevent MitM attacks. In an MitM attack, the attacker makes independent connections with two nodes and relays messages between them, making them believe that they are talking directly to each other over a private connection, when in fact the entire conversation is being controlled by the attacker. The solution to this problem involves that each public key is certified so that the digital signature contained in the certificate guarantees the identification of the user responsible for the public key.

Several models to achieve certification of public keys exist [11]. The most common one is based on a public key infrastructure (PKI), where trust is placed in certificate authorities. Another interesting scheme is based on a decentralized trust model called the web of trust, where each user has a ring with a group of public keys that it trusts. The authors of [12] propose an efficient public key management scheme that is suitable for fully-self-organized MANETs, where all nodes play identical roles. Finally, a certificate-less alternative for PKIs is identity-based cryptography, where the public key of each user is some unique piece of information related to its public identity, so that public-key certificates are unnecessary.

The main disadvantage of asymmetric cryptography is the high computational complexity of the schemes, since most public-key cryptosystems are too heavy to be used in lightweight environments like MANETs in IoT. To solve this issue, this paper proposes a combination of symmetric and asymmetric cryptography to allow the use of secret session keys in these environments. In particular, the proposal offers both strong authentication of legitimate nodes through open communications and the exchange of secret keys shared between pairs, which can be used as session keys.

Node authentication is here performed using an approach based on the idea of zero-knowledge proof (ZKP) [13], which defines a method to prove the knowledge of a certain piece of information without revealing anything about it. Typical ZKPs are based on several challenges and responses, involving a successive exchange of messages, which implies the need to have a stable and continuous connection between nodes [14]. However, this assumption is impossible in a volatile environment like IoT, where sometimes, devices move at a high speed, such as, for example, smart vehicles. In these cases, a massive exchange of messages to run a typical ZKP can be infeasible due to possible connection failures during the protocol. In order to deal with this problem, the idea of non-interactive ZKP (NIZKP) has emerged in the literature [15,16].

In an NIZKP, all of the challenges of a typical ZKP are condensed into a single package sent in a single message. This leads to the optimization of the time necessary for the exchange of messages, so that just a single message is necessary, and even this message can be sent as a beacon in broadcast mode. The work [17] shows a method that transforms an interactive protocol into a non-interactive protocol, which can be applied to turn interactive ZKPs into NIZKPs thanks to the use of hash function. Besides, on the one hand, as a general theoretical result, the authors of [18] present the first NIZKP for NP whose construction is based on one-way permutations and certified trapdoor permutations [19]. On the other hand, as a specific practical result, the work [20] shows an NIZKP of the Hamiltonian cycle problem, which can be used with the proposal described here.

## 3. Preliminaries

If a prover P is trying to prove to a verifier V its knowledge of a solution to a difficult problem, it can use a zero-knowledge proof so that V is not able to trick P and discover the solution or any information that can help it to compute anything faster than before. Thus, what V can see thanks to the protocol should be something that it could have computed by itself. A ZKP must fulfil three main properties, usually called completeness, soundness and zero-knowledge. Completeness means that for any valid input, a prover P can always complete the proof successfully; soundness means that no malicious prover P can construct a valid proof system; and zero-knowledge means that no malicious verifier V is able to derive extra knowledge from the interaction.

This work is based on a non-interactive zero-knowledge proof, which can be formalized as follows. If ${\left\{0,1\right\}}^{*}$ denotes the set of all strings and *R* denotes a witness, for a language $L\subseteq {\left\{0,1\right\}}^{*}$, a pair of probabilistic Turing machines (P,V), in which *P* has probabilistic polynomial time power and *V* has deterministic polynomial time power, is said to be a non-interactive zero-knowledge proof system of the language *L* if it fulfills the following conditions related to correctness and security against malicious provers and verifiers:

- Completeness: For any polynomial p(·) and common input x∈L, *x* is accepted by *V* with a probability greater than 1−ϵ:
  (1) $$Pr\left[V\left(x,R,P\left(x,R\right)\right)=1\right]\geq 1-\frac{1}{p(|x|)}$$
- Soundness: For any interactive Turing machine $P^{\prime }$ representing a dishonest prover, any polynomial p(·) and any common input x∈L provided by $P^{\prime }$ *x* is accepted by *V* with a probability at most *ϵ*:
  (2) $$Pr\left[V\left(x,R,P^{\prime }\left(x,R\right)\right)=1\right]<\frac{1}{P(|x|)}$$
- Zero-knowledge: For any x∈L provided by *P*, no information is revealed from *x* to *V* that it could not compute alone before running the protocol, which means that there is a probabilistic polynomial time algorithm *M*, such that:
  (3) $$V\left(x\right)=x,\left(R\in {0,1}^{c(|x|)},P\left(x,R\right)\right)\approx_{c}M{\left(x\right)}_{x\in L}$$

One of the most important factors of any ZKP is the choice of the mathematical problem that forms its basis. The work [21] showed that, under certain complexity assumptions, on the one hand, any NP problem can be used to define a ZKP, and on the other hand, only problems in BPP (bounded-error probabilistic polynomial time) can be used to describe non-interactive zero-knowledge proofs.

In this work, the chosen problem is the graph isomorphism problem. An isomorphism between two graphs is a bijection that preserves the adjacency relationship, *i.e.*, any two vertices of a graph are adjacent if and only if so are their images in the other graph. The graph isomorphism problem consists of determining whether two graphs are isomorphic or not. This problem has been used in cryptography [22] because an efficient algorithm to solve it in general is yet unknown. In particular, the determination of whether two graphs with the same number *v* of vertices and the same number of edges are isomorphic or not involves a brute force attack, because it requires checking whether some of the v! possible bijections preserve adjacency. In general, the graph isomorphism problem is one of a few problems in computational complexity theory belonging to NP, but not known to belong to a P or to an NP-complete subset [23]. Therefore, its resolution, depending on the size and the type of the involved graphs, can be very difficult. In particular, the graph isomorphism problem has been proven to be in BPP, which opens the door to the definition of NIZKPs based on it.

## 4. NIZKP-Based Authentication

The proposal here described is based on a variant of NIZKP that uses only a single message to verify the knowledge and can be adapted to different levels of security, so that the larger the number of challenges, the higher the level of safety for the verifier. In particular, the parameters used in the proposal are shown in Table 1.

**Table 1:** Proposal parameters.

Notation	Meaning
*G*	Graph known by all legitimate nodes, on which they know how to solve a hard problem
$Sol_{G}$	Solution to the hard problem in *G*
*n*	Number of challenges
$Cha_{i}$	*i*-th challenge proposed by the verifier
$G_{i}$	*i*-th isomorphic graph used as a commitment
$Iso_{i}$	Isomorphism between *G* and $G_{i}$
$Res_{i}$	*i*-th response corresponding to the challenge $Cha_{i}$ on the graph $G_{i}$
h(·)	Cryptographic hash function
LSB(·)	Least significant bit of an input string
$E_{k_{i}}\left(\cdot \right)$	Symmetric encryption with key $k_{i}$
Subkey	Contribution of a node to the session key

In [15], it was shown that the validity of NIZKPs relies on the computational assumption of an ideal cryptographic hash function. Thus, the proposed scheme uses a cryptographic hash function that fulfills such a requirement.

In the described scheme, each node broadcasts a message to identify itself as a legitimate network node. The message consists of a number of commitments defined by isomorphic graphs generated from an initial graph known by all legitimate nodes. For instance, that graph may represent the network, so that each node represents a network user.

Each commitment of the message, except the first one, is encrypted and can only be decrypted after verifying all of the previous ones.

**Figure 1:**
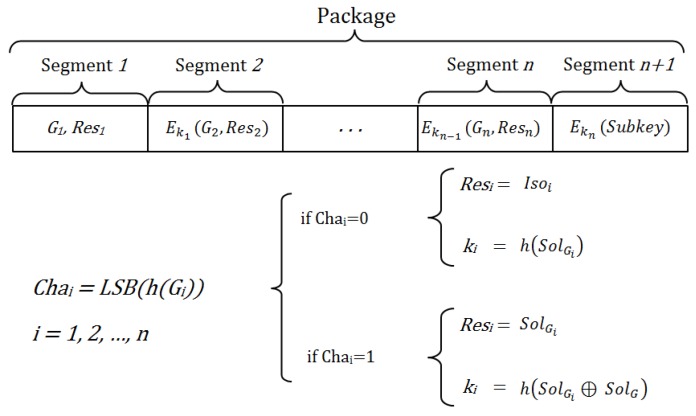
Components of sent messages.

In particular, the message is divided into *n + 1* segments that are all encrypted with different keys, except the first segment, which is not encrypted (see Figure 1). Thus, a legitimate network user can authenticate itself to join a communication session with another node if this latter node is able to decrypt all of the segments of the message broadcast by the first one, so that it can reach the last segment, which contains the contribution of the sender node to the session key.

The encryption key of each segment depends on the previous segment, so that although someone wants to decipher only the last segment, this is impossible, because it would require the decryption of all previous segments. The security level of the scheme depends on the number of segments of the message, which represent different challenges. The greater the number of segments, the more complex it is to reach the last segment and to obtain the information required for the establishment of the shared key.

After the two-way authentication using the same procedure, based on the idea of the Diffie–Hellman scheme, both nodes will know the shared session key, but with both exchanged subkeys.

Each segment, except the last one, contains an isomorphic copy of the original graph. A one-way hash function known by all legitimate network nodes is used to define the challenge that the receiver must solve on each isomorphic graph. Moreover, the same hash function is used to define the encryption key for each message segment.

The operations that the receiver must perform on the message are:

1. Process the first segment of the message, which is not encrypted.
2. Compute, using the hash function, the challenge that matches the information included in the segment.
3. Check whether the response corresponds to the challenge and the isomorphic graph.
4. From the challenge, compute the key it has to use to decrypt the next segment.
5. Apply Steps 2 through 4 until the last segment, which once deciphered contains the information needed to establish the shared secret key.

If the used hash function, problem and graph are adequate, the probability that $Cha_{i}=0$ is 1/2. Thus, the probability that a legitimate node knows the key $k_{1}$ is 1/2, that it knows the two keys $k_{1}$ and $k_{2}$ is $1/2^{2}$,..., and that it knows the *n* keys $k_{1}$, $k_{2}$, ..., $k_{n}$ is $1/2^{n}$.

The challenges have been chosen as the known ZKP is based on isomorphic graphs. However, in the NIZKP here proposed, the challenges are defined from the result of a Boolean output of a hash function defined through the least significant bit (LSB) applied on each committed isomorphic graph. Thus, for each challenge, the response is defined as follows:

- If Challenge=0, the response is the isomorphism.
- If Challenge=1, the response is the solution to the problem in the isomorphic graph.

**Figure 2:**
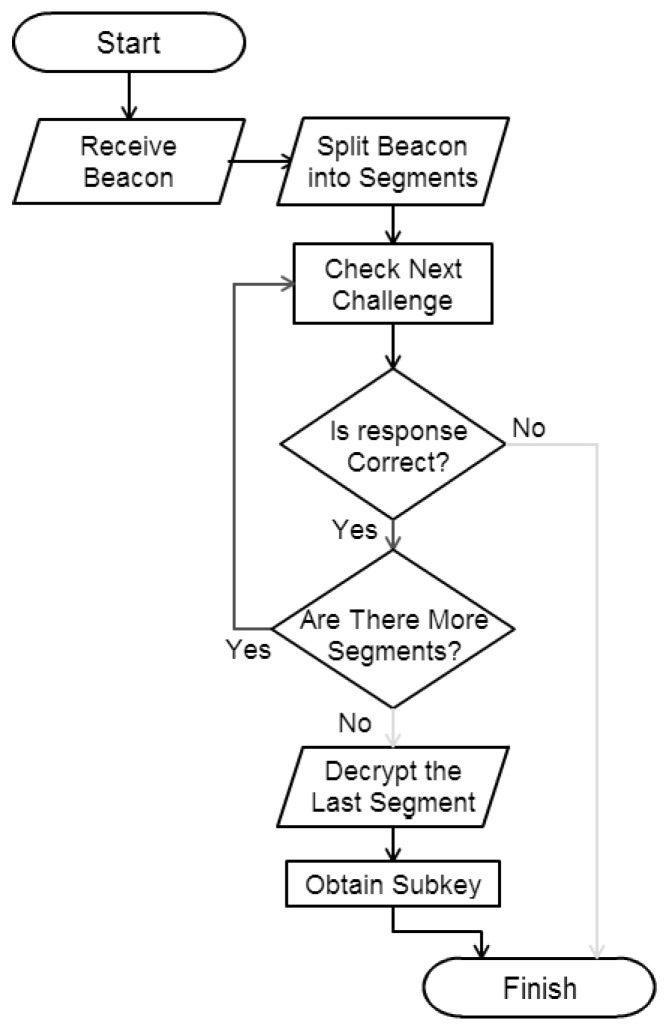
Flowchart of the proposed algorithm.

The flowchart of the algorithm to be run by the receiver is shown in Figure 2. The pseudocode of the proposed scheme (Algorithm 1) is shown below.

**Algorithm 1** Processing*//Params: beacon, encrypted message segments**//Params: t_seg, dimension of beacon segments**//Params: solg, solution in the original graph**//Return: subkey, contribution to the key***function** getData (char[] beacon, int tseg, char[] solg)01: var segs[]; // Stores the message segments02: // Message is divided into tseg−size segments03: segs = beacon.splitByTam(tseg);04: // Isomorphic graph and response05: // First segment is not encrypted06: var gi = getGi(segs[0]);07: var res = getRes(segs[0]);08: // The challenge is computed09: var cha = LSB.hash(gi.getBytes());10: // Check whether the response is correct11: **if** (res != response(gi, cha))12:  return; // If not correct, abort13: **endif**14: // The solution is obtained in gi15: var sol = solve(gi);16: // ki is the encryption key of the next segment17: var ki = $\bar{cha}*hash\left(sol\right)⊕cha*hash\left(sol⊕solg\right)$18: var decryption;19: // The following steps are repeated20: **for** (int i=1;i<segs.size()−1;i++){21:  // The segment is decrypted with the key ki22:  decryption = Crypto.decrypt(segs[*i*], ki);23:  gi = getGi(decryption);24:  res = getRes(decryption);25:  cha = LSB.hash(gi.getBytes());26:  **if** (res != response(gi, cha))27:   return;28:  **endif**29:  sol = solve(gi);30:  ki = $\bar{cha}*hash\left(sol\right)⊕cha*hash\left(sol⊕solg\right)$31: }32: // Decryption of the last segment provides the33: // contribution to the shared key.34: return Crypto.decrypt(segs[segs.size()], ki);**endfunction**

The receiver can access the last message segment thanks to the decryption key obtained from the previous segment after running the algorithm. This last segment contains the contribution of the sending node to the potential session key shared with each receiver.

The initial graph and a secret key that is a solution to a difficult problem in such a graph are known by all legitimate network users. For example, this solution may be a Hamiltonian cycle because the Hamiltonian cycle problem for arbitrary graphs is considered a difficult problem. The Hamiltonian cycle problem consists of determining whether there is a path in the graph that visits each vertex exactly once. This problem is often considered NP-complete, but there are some particular graphs for which the problem is polynomial or even linear. Because of this, in this paper, if the Hamiltonian cycle problem is used, the use of non-planar graphs is suggested. A graph is planar if it can be drawn in a plane without graph edges crossing. In order to check in linear time whether a given graph is non-planar, Theorem 1 can be used:

Theorem 1. For any simple, connected, planar graph with *v* vertices and *e* edges: If v≥3 then e≤3v−6.

Thus, this theorem can be used to prove that a graph is not planar when the above relationship between *v* and *e* is not fulfilled. In fact, when generating the graph, if a planar graph is obtained, edges are added at random until the theorem’s condition is not satisfied, thereby ensuring that the graph is non-planar.

The hash function chosen for computing the challenges and encryption keys for each message segment in the implementation of the proposed scheme is the new standard hash function SHA-3 [24,25].

The symmetric system to encrypt the message segments used in the implementation is the stream cipher of the fourth generation of mobile communications (LTE) [26,27], known as SNOW3G [28]. This choice is based on the linear computational complexity both in encryption and decryption, which guarantees the efficiency and speed of the encryption and decryption processes.

## 5. Applications to the Internet of Things

All of the applications that have been implemented and analyzed in this section to prove the effectiveness of the scheme proposed in this work are for a decentralized environment where Wi-Fi Direct and/or Bluetooth Low Energy are used for wireless communications, because already, many of the interconnected objects have access to these technologies, and they are available for Android. In the future, similar applications will be possibly developed based on the prospective LTE Direct technology.

In particular, the cases in which the required degree of confidentiality is that of communications encrypted with a secret session key are the main use cases of the described system.

The distribution and management of credentials for secure communication can be a relatively simple task if only a restricted group of centralized application providers is considered. Instead, in a distributed architecture, like the one of the Internet of Things, many more problems emerge, as explained in [29], because any device can be connected to any device at any time, and devices might not have had any previous contact with each other in advance. Hence, in this scenario, the problem of key management becomes an important problem. A solution may be based on using a scheme like the proposed one in this paper because it is quite flexible and adaptable to the needs of the devices that interact in IoT.

### 5.1. Applications in Mobile *Ad Hoc* Networks

Conducting business transactions in MANETs is an interesting use case of the proposal, because in that scenario, a legitimate network node might want to share its own resources with other legitimate nodes to carry out such transactions. Due to their mobile nature, often, the nodes of a MANET do not have Internet access in many places. Thus, those legitimate nodes that have an Internet connection may want to rent their connection to other legitimate nodes. For this task, both nodes can establish a shared secret session key, which can be done with the scheme proposed in this paper.

**Figure 3:**
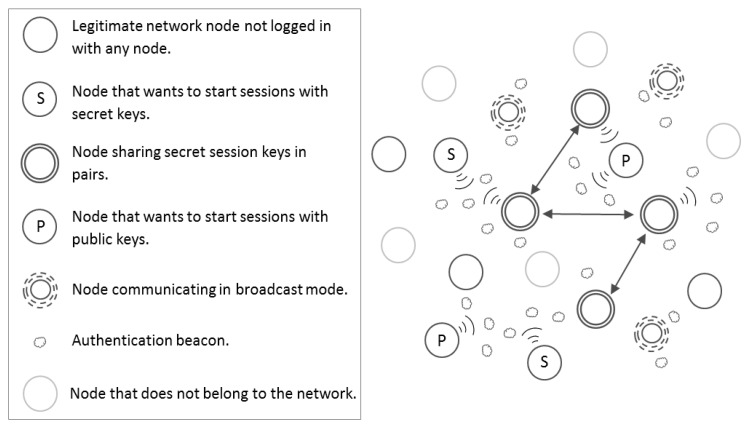
Types of MANET nodes.

In general, two different scenarios for the use of variants of the proposed scheme are described below: To report authenticated information unidirectionally either without using secret keys or using a public key. On the one hand, a node may only want to transmit information from an authenticated way (see Figure 3) so that other legitimate nodes hearing the messages rely on the information that the sending node transmits, thanks to its knowledge of the secret network key used to generate the isomorphic graphs and solutions involved in the described protocol. Two specific examples of use cases within a MANET in this new scenario are event notification or dissemination of advertising shops. The transmission of advertising using the scheme proposed in this paper implies that only legitimate nodes can send advertising, which avoids massive spam from nodes that do not belong to the network. On the other hand, a legitimate node may want to spread its public key in an authenticated way and only to other nodes that also belong to the network (see Figure 3). This use case requires the application of another variant of the proposed scheme where sent beacons hide in their last segment the public key of the sender node. This implies that only legitimate network users can access the public key of the sender hidden in the last segment of the beacon, because the challenges and responses are based on a secret network key. This approach can be used whenever the spread of an event through a MANET requires the use of a digital signature scheme by legitimate nodes, which send their public keys to other legitimate nodes of the network in an authenticated way.

### 5.2. Applications in Vehicular *Ad Hoc* Networks

A vehicular *ad hoc* network (VANET) can be seen as a special type of MANET where the nodes are vehicles and the main objective is to prevent adverse circumstances on the roads and achieve more efficient traffic management.

One of the most important aims in the design of such networks is to resist security attacks [30]. Thus, in the area of VANETs, the proposed scheme can be used to authenticate vehicles in isolated areas (mountain areas, tunnels, *etc.*), where no Internet connection is available. Each vehicle can send an authentication message and agree on a shared key to communicate thereafter, following the proposed scheme based on the Diffie–Hellman idea.

Furthermore, the proposal can be applied to solutions of other problems in VANETs. For example, it can be used to authenticate the information sent from the smart traffic lights to vehicles. Some critical environments require that this information is sent authenticated, so that only legitimate users of the network can decrypt and process it. Thus, for instance, there are proposals like [31] that describes a low-cost solution based on a smart traffic light using a light sensor that provides information in real time about the traffic light color. The solution uses a Bluetooth Low Energy (BLE) module that allows transmitting the state of the traffic light to nearby vehicles, as a beacon notification. This beacon notification can be sent authenticated through the proposal explained in this paper.

### 5.3. Applications in Sensors Networks

Regarding sensor networks, the proposal presented here can be interesting, specifically for its application in wireless sensor networks (WSNs). WSNs have evolved significantly in the last few years, generating a promising research area about a fruitful and useful technology. This technology involves two types of entities: Sensor nodes and base stations. In general, base stations are more powerful than sensor nodes, but this trend is changing thanks to advances in low-powered technology.

Nowadays, it is possible to create a WSN using platforms like Arduino, Rasperry Pi, Odroid, Intel Edison, *etc.*, which can have several sensors. Since some applications in WSNs only require the information of a specific sensor, the scheme proposed in this paper can be used for these platforms to send the sensor information independently and in an authenticated way by using a broadcast beacon to send the data of each sensor. Thus, sent data will only be accessible to legitimate entities.

The applicability of the proposed solution is more to sensor platforms, such as those coordinated through Arduino, than to individual sensors, because Android can be installed in them. Thus, the secure communications based on the proposed scheme can be used on any sensor platform based on Arduino, such as Odroid.

Besides, the proposed authentication scheme can be used to complete other solutions and to add a layer of security. For example, existing WSN models based on preloaded parameters, like broadcast keys for sensor nodes, can be improved by using the scheme presented in this paper to generate shared keys and using them as broadcast keys for the sensor nodes.

## 6. Security Proofs

In the following, several known attacks are analyzed in relation to the proposal.

### 6.1. Attacks on the Cryptographic Operations

On the one hand, the security of the scheme depends on the chosen hash function. A collision attack on a cryptographic hash tries to find two inputs producing the same hash value. Another possible weakness can be a preimage attack, which tries to find a message that has a specific hash value. A cryptographic hash function should resist both preimage and collision attacks. In this work, the implementation uses the hash function SHA-3 for several operations. The algorithm behind SHA-3 is the Keccak function. With Keccak, it is possible to target a given security strength level by choosing the appropriate capacity, *i.e.*, for a given capacity *c*, Keccak is claimed to resist any attack up to complexity $2^{\frac{c}{2}}$. This approach is similar to the one suggested to choose the security strength used in [32] by NIST (National Institute of Standards and Technology).

On the other hand, the security of the proposal depends on the symmetric cipher used to encrypt each segment. The encryption used in the implementation is the basis of the security in LTE communications, called SNOW 3G. The SNOW 3G core is supplied as portable Verilog, with a Verilog Hardware Description Language (VHDL) version available, thus allowing customers to carry out an internal code review to ensure its security.

Therefore, the security of cryptographic operations is guaranteed due to the used standards: SHA-3 and SNOW 3G. The SHA-3 algorithm is based on a permutation where collisions and preimages can be found for its cryptographic hash function in one query to the permutation. The sponge construction has been proven to be indifferentiable from a random oracle if the underlying permutation is assumed to be ideal [33], and this result applies to Keccak function. Optimal bounds have been obtained on collision resistance and on preimage and second preimage resistance for Keccak in the ideal permutation model [34]. Regarding SNOW 3G, it has linear time complexity, which guarantees efficiency during the encryption/decryption process. Furthermore, security proofs of SNOW 3G are based on the assumption that this encryption system behaves like a perfect random function of the key [35].

### 6.2. Attacks on the Graph Problem

The specific problem under the ZKP is fundamental for the security of the scheme. The implemented scheme uses the two graph problems of the graph isomorphism and of the Hamiltonian cycle.

Regarding possible attacks to the problem of the graph isomorphism, some efficient algorithms for some specific graphs have been proposed. For example, in [36], several probabilistic algorithms were discussed; and in [37], some algorithms for determining whether two graphs are isomorphic or not have been described. However, the proposed scheme can use graphs of different sizes, and depending on the size of these graphs, the security can be increased. The generated graphs are completely random, and therefore, none of the proposed algorithms are useful for finding the secret. Therefore, under the hypothesis of using well-chosen instances of the problem, the graph isomorphism problem can be considered NP-complete for the particular execution of the proposal.

With respect to the Hamiltonian cycle problem, under the condition of the defined scheme, the problem can be also considered NP-complete. This is achieved with the use of non-planar graphs, because through the theorem shown in a previous section, we can ensure that in the implementation, none of the graphs that are generated in the scheme are planar. Again, under the hypothesis of using well-chosen instances of the problem, the Hamiltonian cycle problem can be considered NP-complete for the particular execution of the proposal. Thus, a real attack based on the used graph problems is not possible because the complexity of the chosen problems can be considered NP-complete.

### 6.3. Man in the Middle Attack

An MitM attack happens when an attacker secretly relays and possibly alters the communication between two parties who believe they are directly communicating with each other. This is one of the most used attack schemes in wireless networks.

In the case of the proposal, an MitM attack cannot be carried out because there is no way to gain information during the transaction. In particular, the scheme uses a single beacon to send some information from the sender to the receiver. Therefore, there is no communication between them, so that other users cannot intercept communication and, hence, cannot impersonate communications.

If the scheme is used to establish a secret key through the Diffie–Hellman algorithm, thanks to the fact that the protocol uses strong mutual authentication with secret keys, it is robust against an MitM attack. Mutual authentication refers to two parties authenticating each other at the same time. Consequently, an MitM attack against the proposed scheme would not succeed. The attacker could intercept beacons, but without access to secret parameters of the scheme, it cannot get any confidential information. The configuration parameters of the proposed scheme are only accessible for the legitimate users of the network and are provided by a trusted third party during the initialization of the scheme.

### 6.4. Other Attacks

In MANETs, due to the lack of a centralized structure, denial of service (DoS) attacks can be frequent. In order to protect the proposed scheme from DoS attacks, although the communication produced with the application is through a non-secure channel, only legitimate nodes of the networks are able to send and decrypt valid messages.

The proposed scheme is resistant against a brute force attack. A brute force attack, also called exhaustive key search, is a trial-and-error method used to obtain relevant data through the generation of a large number of consecutive guesses about the desired data. In the proposal described in this paper, since the desired data are contained in the last segment of the sent package, a brute force attack on the key used to encrypt the last segment would be required. In order to make it possible that a brute force attack could be used to discover the key used to encrypt such a segment, it could be necessary to check all of the possible keys. The key used to encrypt each segment has a fixed size defined by the hash function used to obfuscate the response to the previous segment challenge. SHA-3 has been used as a hash function in the implementation, so since its smallest output is 224 bits, a brute force attack would involve checking $2^{224}$ combinations. If the scheme is used as a key agreement protocol, since in that case, interaction between parties exists, a maximum response time could be defined in order to prevent possible brute-force attacks.

Another dangerous attack in MANETs is the sibling attack, which occurs when a node illegitimately uses multiple identities. This problem is avoided in the proposed scheme thanks to the distributed nature of the used NIZKP.

Finally, the proposal is also resistant to identity theft because node access is controlled by an NIZKP.

In a nutshell, usual attacks have no harmful effect on the proposal, because its security is supported by NIZKP based on complex mathematical problems and current standard hashing and encryption.

## 7. Implementation

The described scheme is intended for its use in areas related to the Internet of Things. It has been implemented for Android and Android Wear platforms, which are two examples of the proliferation of devices in this new dimension of the Internet. Android is the most popular smartphone operating system, with over 80% market share worldwide. Android Wear is the operating system based on Android for wearable devices of the same company and has more than 90% of the market share in the devices of its kind. Therefore, all of the results presented here are the result of the implementation of the scheme in these two platforms belonging to the Android Open Source Project. The source code is open source under a Git repository on the GitHub Platform [38].

**Figure 4:**
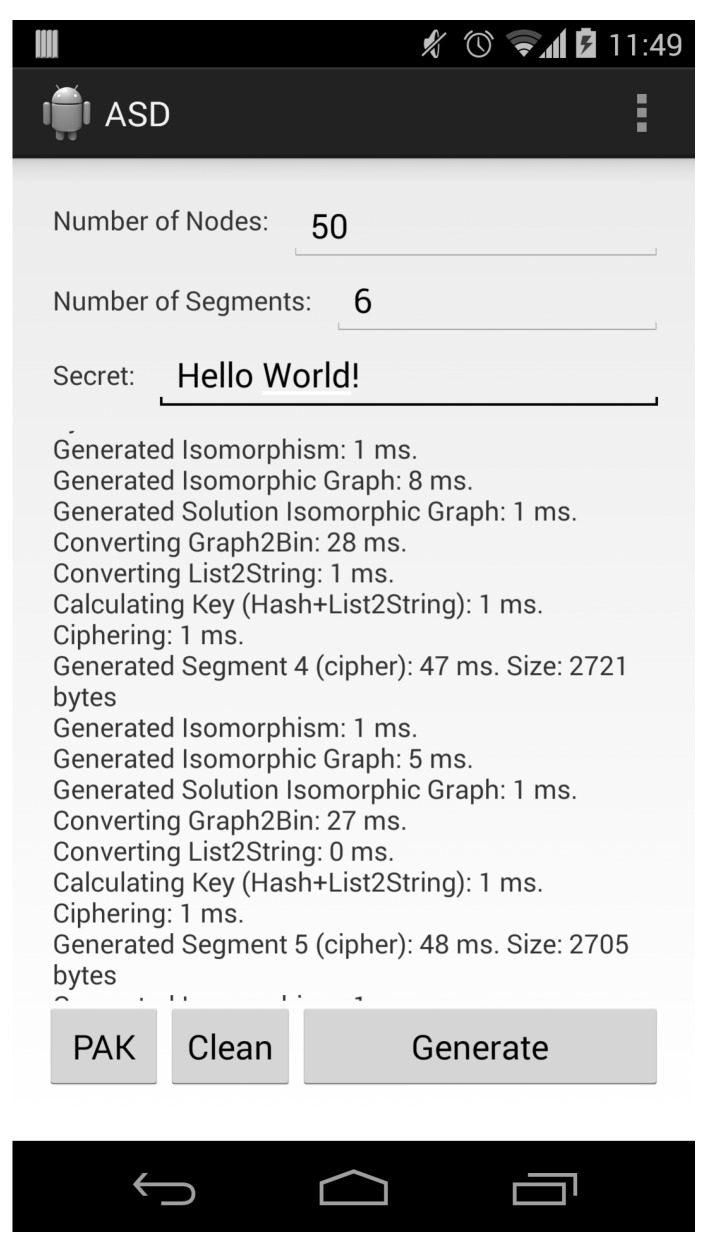
Android application screenshot.

Figure 4 shows a screenshot of the Android application that was created to analyze the performance of the scheme in smartphones.

Despite the implementation described in this section having been focused on IoT devices where it is possible to have an Android operating system, like current smartphones and smartwatches, this does not mean that the necessary devices have to be extremely powerful devices. For instance, there are sensor platforms, like Odroid, that, despite their small size, are able to run an operating system like Android, so consequently, they can be used to run the proposed system. Besides, Google has recently introduced a new operating system for IoT, called Brillo, which is a fork of Android, so the implementation described in this paper is easily adaptable to future devices with the Brillo operating system.

### 7.1. Package Format

One of the premises that the scheme meets is that it is lightweight in terms of size and the speed of computation, so it fulfills the requirements of the devices of the IoT. Therefore, the size of the message or package has been reduced in the implementation to be as small as possible in order to use the smallest possible space in memory and in order to have fast communication between devices. Because of this, also the storage format of each of the elements that make up the message has been optimized. Thus, the following measures have been taken to serialize these elements:

- Graphs: Graphs are serialized by denoting their adjacency matrices into integers, because this is their fastest implementation. This serialization has been also improved by using hexadecimal rather than integer numbers. In this case, the storage size is smaller, but the speed serialization is quite slow. Thus, a faster serialization has been chosen compared to a slight improvement in the package size, so graphs have been serialized through their adjacency matrices.
  For instance, given the adjacency matrix:
  $$\left(\begin{array}{ccccc} 0 & 1 & 1 & 0 & 1\\ 1 & 0 & 1 & 0 & 0\\ 1 & 1 & 0 & 0 & 1\\ 0 & 0 & 0 & 0 & 1\\ 1 & 0 & 1 & 1 & 0 \end{array}\right)$$
  it can be represented in one dimension as:
0110110100110010000110110
  This one-dimensional representation is serialized by converting it into an integer of eight digits, formed of five integers separated by the character “,”
  13,20,25,1,22
- Solutions: Solutions have been serialized by using lists of integers separated by the character “,” because both types of solutions, the Hamiltonian cycle and the isomorphism between graphs, can be represented as lists. For example, if the isomorphism between two graphs is 1→2, 2→5, 3→1, 4→3, 5→4, using array indexes as original nodes and the values of the array as the values of the new nodes, the isomorphism is represented as:
  2,5,1,3,4
- Segments: Each segment of the message is serialized in hexadecimal. Besides, all of the segments, but the first one, are encrypted as explained in previous sections. The last segment contains a secret encrypted by the key that can be obtained with the solution to the challenge of the previous segment. For instance, the aspect of a segment containing an isomorphic graph and challenge and response corresponding to the graph is the following, expressed with hexadecimal characters:
  A324D0E3F19
- Message or package: The complete message is the concatenation of all of the generated segments. To separate the segments, the character “|” is used. An example of a package with three segments is:
  A324D0E3F19|F9223B3EE34|DC34F212ACB

### 7.2. Package Size

Given the format that has been used to represent the elements that are part of the proposed scheme, optimal package sizes have been achieved.

The message size is given by the dimension of the graph used to represent the network. The more nodes a graph has, the more space is needed. This involves the segments being larger, resulting in larger package sizes.

**Figure 5:**
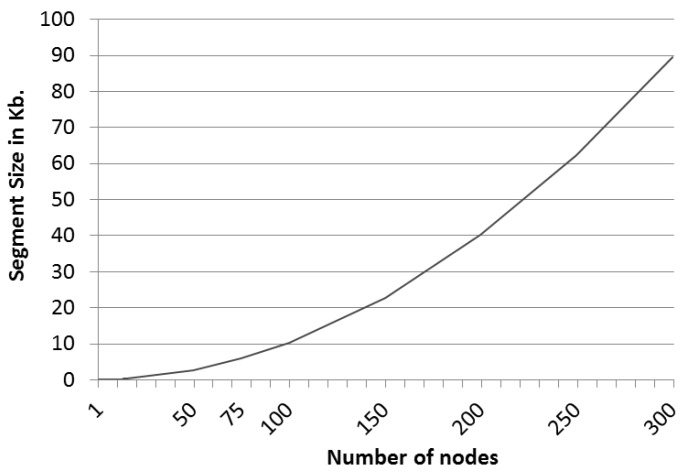
Segment size trend.

Therefore, we have analyzed which sizes per segment are recommended depending on the number of nodes in the graph, which are shown in Figure 5.

This clearly reveals a polynomial tendency of order two that represents the relationship between the size of the segments and the number of nodes of the graph that represents the network. Thus, the following polynomial function (see Equation (4)) has been calculated that defines the data, so that segment sizes for graphs with more nodes can be estimated.

(4) $$y=0.9765x^{2}+5.8046x-1.1513$$

Thus, for instance, a scheme in which the graph of the network is defined by 50 nodes and the number of challenges that defines the scheme package is six would require a package size of 16 kilobytes without including the size of the shared secret. Thus, in order to send a message in a single package securely by using this scheme, we have used six challenges for 50 nodes, and the sent message has an overhead of only 16 kilobytes.

### 7.3. Package Generation Time

The computational time required for the devices to create the packages to send depends on the number of segments that are defined in the package. Thus, the larger the number of segments, the greater its security and the more time that the microprocessor of the device requires for generating the package.

For the tests, smartphones have been chosen from three possible ranges, low-cost, mid-cost and high-cost, and being several years old. The selected smartphones models are: Motorola Moto G, Samsung Galaxy S3 and LG Nexus 5. This selection has been taken to verify the effectiveness of the scheme on devices with limited computing capacity.

Furthermore, Android Wear smartwatches have been also used for testing, specifically the LG G Watch and Samsung Gear Live models. Since the smartwatches depend on a smartphone, which must be constantly connected via Bluetooth Low Energy, package generation is in fact done from the smartphone.

**Figure 6:**
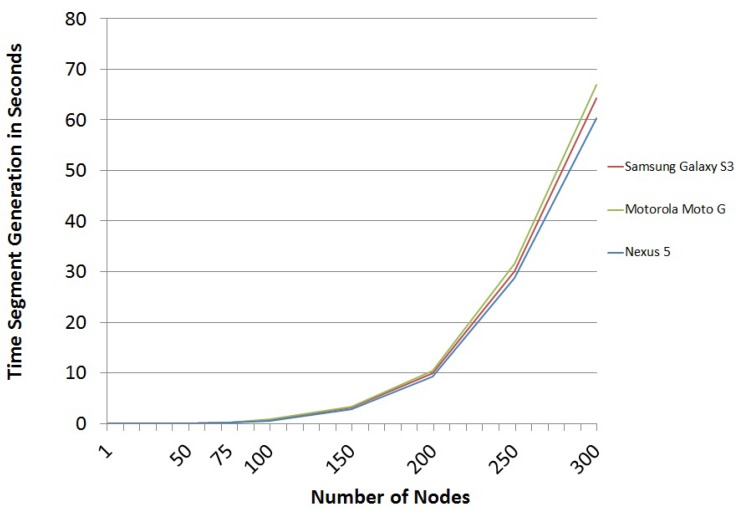
Segment generation time trend

Considering all of these characteristics, the programming language that has been used is Java, because it is the most widely-used platform for programming in the Android Open Source Project. After making dozens of experiments, the average of the results is as shown below. The time for segment generation based on the number of nodes in the graph is shown in Figure 6.

However, the results shown in Figure 6 are strongly conditioned by the process of the serialization of graphs. In fact, this process requires much more computation time than the sum of all of the remaining steps of the scheme. Not surprisingly, the serialization process for large graphs requires more than 98% of the time required to build the package. The first steps of the serialization process were much slower, more than 20 times, before the adjacency matrix was converted into hexadecimal characters. However, after optimizing the process, it was concluded that it is more efficient to convert it into integers using the characteristics of Java classes. The problem is that now, the occupied space is greater than in the previous process. The computational speed has improved by a factor of 20, but still, the process remains very slow compared to other operations of the scheme, due to the limitations of the Java Virtual Machine. For example, for a network of 300 nodes, a graph serialization takes about 59,138 ms. The complete generation of a segment, including the serialization graph, takes about 60,345 ms. These results can be substantially improved by implementing the scheme in a low-level language that Android Open Source Project has, such as the programming language C, which is a compiled, not interpreted, language like Java.

From the results of Figure 6, the trend has been obtained, and the associated polynomial of order four has been calculated (see Equation (5)), which represents the number of nodes and the segment generation time. This equation can be seen as an estimation of how long it would take to create packages for testing, depending on the number of nodes of the graph.

(5) $$\begin{array}{c} y=\left(5e-6\right)x^{4}+\left(23e-4\right)x^{3}-0.537x^{2}+30.548x-227.48 \end{array}$$

For example, in a scheme where the graph is defined by 50 nodes and the number of challenges that defines the scheme package is six, the time to build the package is 300 ms. Thus, a mobile device with limited capabilities could easily generate packages for the proposed scheme, as demonstrated in these implementations.

### 7.4. Package Processing Time

**Figure 7:**
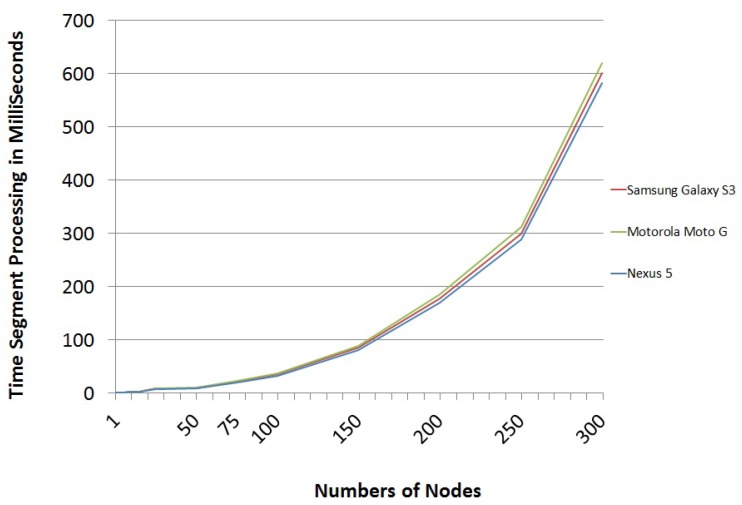
Segment processing time trend.

Finally, we have analyzed the time it would take mobile devices to decipher the package that comes from other mobile devices. These package-processing times are really short, as evidenced by the processing time per segment shown in Figure 7.

After analyzing the above results, it was concluded that the trend of the data follows a polynomial function of order four (see Equation (6)). This equation can be used to estimate the processing time per segment depending on the nodes of the graph representing the network.

(6) $$y=\left(2E-9\right)x^{5}\left(-1E-6\right)x^{4}+\left(2E-4\right)x^{3}-0.0184x^{2}+0.673x-1.7392$$

This means that, for instance, a scheme in which the graph of the network is defined by 50 nodes and the number of challenges that defines the scheme package is six, the time a mobile device takes to process a received package to get the secret is only 48 ms.

## 8. Comparative Analysis

In the recent literature, it is hard to find novel schemes proposed for the authentication of mobile devices, specifically in the Internet of Things.

Many existing research works propose lightweight authentication schemes based on challenges and responses, but none of the proposed schemes have been implemented completely. Many authors omit one of the most important pieces of information that can characterize a scheme of this kind: the time required for authentication. Just a few authors have analyzed the transmission bit rate, the percentage of packages that are lost in interactive schemes and/or the total real time that schemes require. In this work, those parameters are studied, but the percentage of lost packages is not analyzed, because the scheme is non-interactive and just one single message is sent, so no loss can happen.

### 8.1. ZKP Schemes

We have analyzed several lightweight authentication schemes applied to the Internet of Things. For instance, [39] proposes a zero-knowledge proof authentication algorithm based on isomorphic graphs and describes its evaluation and implementation. The proposed mechanism allows authentication with varying confidence and security levels. Such a work describes an implementation on conventional computers (with different configurations), so that the starting conditions are more powerful than when using mobile devices, as in this paper, mainly because of the efficiency of the used programming language. On the one hand, the scheme proposed in this paper uses Java as a programming language, so that the implementation is as cross-platform as possible and, thus, can be applied to many current mobile devices. On the other hand, the compared scheme uses random graphs of 41 nodes. In order to allow the comparison, the scheme proposed in this paper has been also implemented with graphs of 41 nodes. The comparison results are shown in Table 2, where the four different hardware configurations described in the paper are shown. Another consideration is the fact that the compared scheme uses an interactive ZKP with six exchanges of messages, while the proposal of this paper is based on an NIZKP in which only one single message is necessary.

**Table 2:** Comparative data: time (ms) and size (bytes).

		Conf1. [39]	Conf2. [39]	Conf3. [39]	Conf4. [39]	Our Scheme
10 Challenges	Time Size	469 4045	1302 4045	484 4045	1522 4045	454 17,826
100 Challenges	Time Size	3422 39,595	8070 39,595	3703 39,595	9824 39,595	5665 187,132

From Table 2, it can be concluded that the scheme that has been designed here is computationally faster considering the average results and characteristics of both implementations. On the other hand, the compared scheme uses fewer memory bytes. However, the relationship between memory and time is better in the scheme proposed here than in the others. This is due to the fact that the difference in memory space of a few kilobytes is acceptable for today’s mobile devices, both when being sent with wireless technologies and when being stored and processed.

### 8.2. Schemes Based on Diffie–Hellman

Other schemes with an objective similar to that of the one proposed here have been also compared. One of them was chosen for this study due to its popularity, frequent application and use of Diffie–Hellman key exchange [6]. Such a method allows two parties that have no prior knowledge of each other to jointly establish a shared secret key over an insecure communication channel, so that this key can then be used to encrypt subsequent communications using a symmetric key cipher. However, if no additional security measure is used, the Diffie–Hellman key exchange is vulnerable to an MitM attack. This vulnerability is possible when Diffie–Hellman key exchange does not include the authentication of participants. However, feasible solutions for authenticated Diffie–Hellman schemes, such as the one proposed here, allow preventing such attacks.

After reviewing the relevant bibliography, a scheme was chosen that implements an authenticated Diffie–Hellman protocol for the comparison. The scheme is called password authenticated key (PAK) Diffie–Hellman exchange [40] and proposes to add mutual authentication based on a memorizable password, to the basic, unauthenticated Diffie–Hellman key exchange. PAK allows two parties to authenticate themselves while performing the Diffie–Hellman exchange. It also provides a secure and authenticated key-exchange protocol, which ensures forward secrecy and is secure against offline dictionary attacks when passwords are used.

For the comparison, the PAK scheme was implemented for Android and Android Wear devices. Till now, the PAK scheme had not been implemented on any Android platform, so this paper provides the first implementation of the PAK scheme in the most popular platform of mobile devices. Both the source code of the scheme proposed in this paper and the source code for the PAK scheme have been released as open source and are available in a hosted repository on GitHub [38]. In the tests, the shared secret information in both schemes has been the typical ’Hello World!’ used in computer programming environments.

In particular, in order to compare the proposed scheme with the PAK scheme, the following configuration was used:

- Graphs of 41 nodes, which are large enough to be considered secure in the scheme; thus, an attack on the graph isomorphism would involve 41! iterations, which would be unfeasible.
- Packages with 3, 4, 5, 6, 7 and 8 challenges.
- The time for these comparisons includes both package generation by the sender and unpacking the package by the user who receives it.

The results of the comparison are shown in Table 3. The conclusion is that in general, the scheme proposed in this paper has a similar performance as the PAK scheme, and even in some cases, the proposed scheme slightly improves the results of the PAK scheme.

**Table 3:** Password authenticated key (PAK) scheme *vs.* the proposed scheme.

PAK Scheme	Our Scheme	
Time (ms)	Challenges	Time (ms)
197	3	86
	4	112
	5	153
	6	176
	7	195
	8	221

## 9. Conclusions

With the proliferation of electronic devices in many areas, a new paradigm called the Internet of Things has emerged. One of the biggest threats to the deployment of the networks involved in the Internet of Things is the security of communications, so new lightweight cryptographic algorithms that are adapted to the computing capabilities of mobile devices are necessary. In this paper, a novel scheme based on the idea of non-interactive zero-knowledge proofs is presented. The proposal only requires sending a message to share confidential information in an authenticated way. Thus, the new algorithm is proposed to be used for establishing authenticated secret session keys between pairs of legitimate nodes of mobile *ad hoc* networks. Furthermore, the scheme can be used by nodes that want to send authenticated information in broadcast mode to other legitimate nodes in the network. Different use cases are defined for the authenticated public key exchange in these networks by using variants of the scheme described in this paper. The proposal is mainly characterized by not using interaction for authentication, which is very adequate for volatile networks, such as those in the Internet of Things, which require a more efficient authentication procedure. The scheme has been implemented for the platforms under the seal of the Android Open Source Project, and the design has been optimized to reach a high level of security at a low cost of memory consumption. The obtained results are promising, as they improve the performance of other lightweight authentication schemes based on zero knowledge proofs. Several open problems still remain and could be solved in future work, such as the design of an improved service system based on the proposal.
